# The ATM signaling network in development and disease

**DOI:** 10.3389/fgene.2013.00037

**Published:** 2013-03-25

**Authors:** Travis H. Stracker, Ignasi Roig, Philip A. Knobel, Marko Marjanović

**Affiliations:** ^1^Oncology Programme, Institute for Research in Biomedicine (IRB Barcelona)Barcelona, Spain; ^2^Departament de Biologia Cellular, Fisiologia i Immunologia, Institut de Biotecnologia i Biomedicina, Universitat Autònoma de BarcelonBarcelona, Spain

**Keywords:** ataxia-telangiectasia, Nijmegen breakage syndrome, AT like disease, ATM, Mre11 complex, apoptosis, senescence, DNA repair

## Abstract

The DNA damage response (DDR) rapidly recognizes DNA lesions and initiates the appropriate cellular programs to maintain genome integrity. This includes the coordination of cell cycle checkpoints, transcription, translation, DNA repair, metabolism, and cell fate decisions, such as apoptosis or senescence (Jackson and Bartek, 2009). DNA double-strand breaks (DSBs) represent one of the most cytotoxic DNA lesions and defects in their metabolism underlie many human hereditary diseases characterized by genomic instability (Stracker and Petrini, 2011; McKinnon, 2012). Patients with hereditary defects in the DDR display defects in development, particularly affecting the central nervous system, the immune system and the germline, as well as aberrant metabolic regulation and cancer predisposition. Central to the DDR to DSBs is the ataxia-telangiectasia mutated (ATM) kinase, a master controller of signal transduction. Understanding how ATM signaling regulates various aspects of the DDR and its roles *in vivo* is critical for our understanding of human disease, its diagnosis and its treatment. This review will describe the general roles of ATM signaling and highlight some recent advances that have shed light on the diverse roles of ATM and related proteins in human disease.

## THE DNA DAMAGE RESPONSE TO DOUBLE-STRAND BREAKS

**FIGURE 1:**
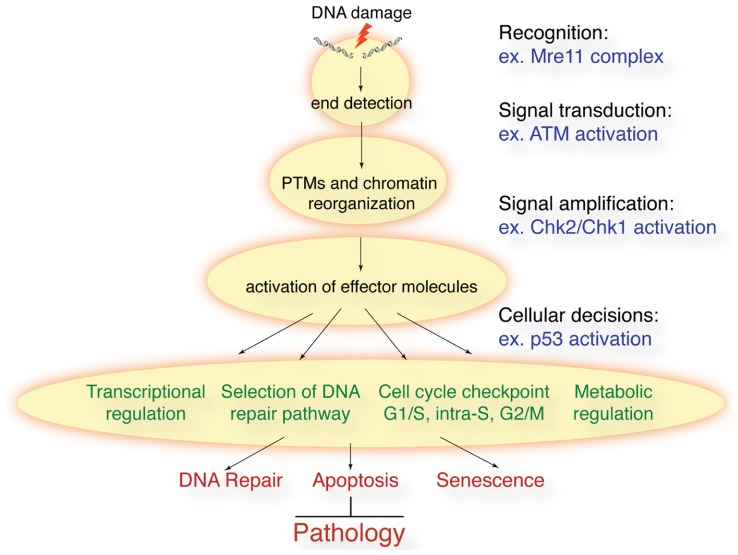
**Schematic of the DDR to DSBs**. Following DSB induction, they are recognized by sensor proteins such as the Mre11 complex. This leads to the activation of ATM and related kinases that promote rapid post-translational modifications (PTMs) on many proteins and remodeling of chromatin structure around the break sites. Effector proteins such as the Chk1 and Chk2 kinases amplify the signal and cells can activate cell cycle checkpoints, regulate transcription, translation, and metabolism, and activate the appropriate DNA repair processes. In some cellular contexts, or in the face of irreparable lesions, cells can activate apoptosis and senescence. The collective result is the prevention of genomic instability and the accompanying pathological outcomes.

In response to a diverse array of DNA lesions, cells mount a DNA damage response (DDR) to maintain genome integrity (Jackson and Bartek, 2009). Following the recognition of a DNA lesion by a sensor protein, the DDR sets in to motion a complex network of signal transduction. The DDR (**Figure 1**) controls cell cycle checkpoints, regulates transcription, recruits the appropriate DNA repair machinery to lesions, responds to metabolic requirements, and controls cell fate decisions, such as apoptosis and senescence. Ultimately, the DDR will prevent genomic instability from accumulating by preventing cells with damaged DNA from propagating or being passed on to progeny through the germline.

While cells must identify and respond to diverse lesions, the DNA double-strand break (DSB) represents a particularly important threat to genome integrity. DSBs can be generated by exposure to ionizing radiation (IR) or various chemical compounds, such as topoisomerase inhibitors, that interfere with DNA replication and cell division. More pertinent to the developmental pathologies of hereditary diseases arising from deficiencies in the DDR are endogenous sources of DSBs. The generation of the antibody repertoire as well as the maturation of germ cells both involve the programed generation and repair of DSBs via cellular enzymes (Nussenzweig and Nussenzweig, 2010; Sasaki et al., 2010). DSBs can also arise during DNA replication due to exposure to metabolites, such as reactive oxygen species (ROS), the activity of enzymes, such as topoisomerases, which break and rejoin DNA strands, and limitations in raw material needed for replication, such as nucleotides, that can promote fragile site expression and chromosomal breakage (Tsantoulis et al., 2008; Bester et al., 2011). Two major pathways of DSB are utilized in the cell, non-homologous end-joining (NHEJ), that is operative throughout the cell cycle, and homology directed repair (HDR) that is restricted to S/G2 when a sister chromatid is present as a template (for a detailed overview of these repair pathways and subpathways, we refer the reader to a recent review; Chapman et al., 2012). The killing of cancer cells via DSB generation is a major strategy in cancer treatment and the cellular responses and mechanisms of repair and acquired resistance to these agents is important to understand in order to improve the efficacy of current treatment regimens (Helleday et al., 2008).

## DNA DOUBLE-STRAND BREAKS AND HUMAN DISEASE

The identification of numerous human genetic instability syndromes, as well as their modeling in different experimental systems, has been invaluable to our understanding of the DDR in human disease. Ataxia-telangiectasia (A-T, *ATM* mutation), the related A-T like disease (ATLD, *MRE11* mutation), Nijmegen breakage syndrome (NBS, *NBS1/NBN* mutation) and the more recently identified NBS like disease (NBSLD, *RAD50* mutation), all present with similar pathological outcomes in humans (Stracker and Petrini, 2011). Cells from these patients have increased levels of chromosomal instability, are highly sensitive to DSBs, and show defective signaling responses such as impaired checkpoint activation or variable defects in apoptosis. Patients are particularly affected in central nervous system (CNS) development, exhibiting either neurodegeneration or microcephaly, and display varying degrees of immunodeficiency (McKinnon, 2012). In addition, these disorders are often characterized by cancer predisposition and in some cases extensive problems related to fertility and metabolism. This review will focus on ATM kinase signaling and attempt to highlight recent work that has improved our understanding of its role in human disease through the regulation of DSB signaling and additional cellular functions that extend beyond the DDR.

## ACTIVATION OF THE ATM KINASE: A CENTRAL TRANSDUCER OF DSB SIGNALING

### ACTIVATION IN RESPONSE TO DNA DOUBLE-STRAND BREAKS

Double-strand breaks are recognized by the Mre11–Rad50–Nbs1 (MRN) or Mre11 complex, which is a sensor of DSBs. Capture of DNA ends by the Mre11 complex leads to the rapid activation of the ataxia-telangiectasia mutated (ATM) kinase (Stracker and Petrini, 2011). ATM is a member of the phosphatidylinositol 3-kinase-related kinase (PIKK) family and is the primary transducer of DSB-induced signaling (Lempiainen and Halazonetis, 2009). The closely related disease pathology resulting from mutations in ATM, or any of the Mre11 complex genes, highlights their intimate relationship in DSB signaling. However, it is also worth noting that both ATM and the Mre11 complex have central functions independent from one another as ATM is synthetically lethal with many hypomorphic mutations in the Mre11 complex, some of which do not impair ATM activation (Williams et al., 2002; Theunissen et al., 2003).

**FIGURE 2:**
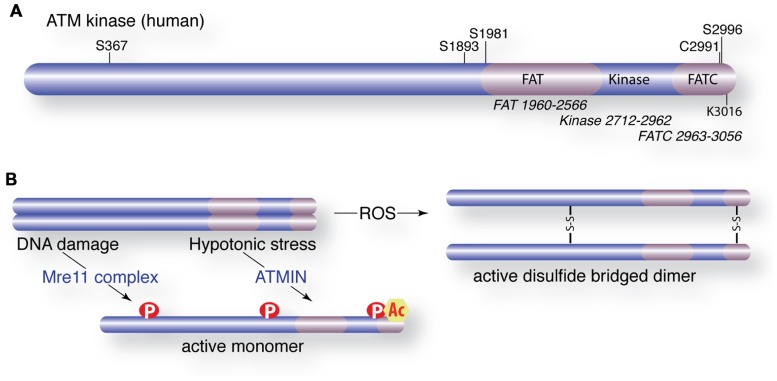
**Activation of ATM and post-translational modifications**. **(A)** Schematic of the ATM protein with domain organization (FAT = FRAP, ATM, and TRAP). Major autophosphorylation sites (S367, S1893, S1981, S2996), the TIP60 acetylation site (K3016) and a critical cysteine involved in ROS activation are shown. **(B)** Activation of ATM by DNA damage or hypotonic stress requires the Mre11 complex (Mre11, Rad50, Nbs1) or ATMIN, respectively. Activated ATM is monomeric, phosphorylated and acetylated. Alternatively, ATM is activated directly by ROS that oxidizes cysteine residues to promote disulfide bridge-mediated dimerization.

In undamaged cells, ATM exists in a dimeric or multimeric configuration (Bakkenist and Kastan, 2003). Following Mre11 complex sensing of DSBs, ATM undergoes autophosphorylation on at least four residues (S367, S1893, S1981, and S2996) that promote its monomerization and kinase activity (**Figures 2A,B**; Bakkenist and Kastan, 2003; Kozlov et al., 2006, 2011). Autophosphorylation is regulated through interactions with several phosphatases that exert opposing influences, including protein phosphatase 2A (PP2A), protein phosphatase 5 (PP5), and wild type p53-induced phosphatase 1 (WIP1; Ali et al., 2004; Goodarzi et al., 2004; Shreeram et al., 2006a). Human ATM-deficient cells complemented with S1981A, S367A, or S2996A mutants showed defective ATM-dependent responses to DNA damage (Kozlov et al., 2011). However, a murine allele with a triple mutation in the analogous sites to human S367, S1889, and S1981 complemented the defects of ATM deficiency *in vivo*, including checkpoint activity, germ cell development, lymphocyte development, and radiosensitivity (Daniel et al., 2008). Furthermore, autophosphorylation is not required for the activation of ATM in several *in vitro* settings (Lee and Paull, 2005; Dupre et al., 2006). The reasons for these discrepancies between complementation experiments with human and murine ATM remain unclear but may reflect species-specific differences or the experimental context (a recent review from the Khanna and Lavin groups included a detailed discussion of this issue; Bhatti et al., 2011).

The modulation of chromatin structure influences radiosensitivity and it has become clear that chromatin status plays a major role in ATM activation and the regulation of its activity at break sites (Bakkenist and Kastan, 2003; Murga et al., 2007). Acetylation of lysine 3016 by the TIP60 acetyltransferase is required for ATM activation in response to DSBs (Sun et al., 2005). TIP60 activity is dependent on activating transcription factor 2 (ATF2) and an interaction with histone H3 lysine 9 tri-methylation (H3K9me3) that is unmasked following the damage-induced removal of heterochromatin protein 1b (HP1b; Bhoumik et al., 2005; Sun et al., 2009). The Mre11 complex is crucial for the localization of TIP60 to H3K9me3, a well-established marker of heterochromatin that also occurs in other regions of the genome. ATF2 interacts with TIP60 and independently controls its levels and activity prior to damage. Dissociation of ATF2 following damage results in higher levels and activity of TIP60 to promote ATM activation (Bhoumik et al., 2008).

In addition to promoting ATM activation, the Mre11 complex, in conjunction with mediator of DNA damage checkpoint protein 1 (MDC1) mediator protein and the high mobility group protein HMGN1 (high mobility group nucleosome binding domain 1), facilitates the chromatin retention of ATM following DSB detection. The Mre11 complex promotes the rapid loading of active, phosphorylated ATM at breaks through interactions with the C-terminus of Nbs1 (Falck et al., 2005; You et al., 2005, 2007). ATM interactions with chromatin are enhanced in cells treated with histone deacetylase inhibitors or in cells lacking HMGN1, arguing that chromatin topology plays a crucial role in mediating ATM–chromatin interactions, even in the absence of DSBs (Kim et al., 2009). Despite enhanced chromatin retention of ATM in cells lacking HMGN1, DSB-induced activation of ATM is impaired, consistent with the proposition that ATM interactions with chromatin prior to DNA damage govern its activation.

### POST-TRANSLATIONAL MODIFICATIONS OF ATM

The mechanism by which post-translational modifications (PTMs) regulate ATM activation and activity remains largely unclear. It has been proposed that autophosphorylation sites may have cell cycle-specific roles or regulate other modifications that are required for ATM activation (Kozlov et al., 2011). The phosphorylation of ATM at S794 by cyclin-dependent kinase 5 (CDK5) in post-mitotic neurons has been reported to be required for subsequent autophosphorylation of S1981, providing some precedence for sequential regulation of ATM (Tian et al., 2009). The N-terminus of ATM has been identified as an interaction domain with Nbs1 and is required for chromatin retention (Falck et al., 2005; Young et al., 2005). Different patterns of phosphorylation in the N-terminus could modulate substrate interactions, chromatin binding, or subcellular localization. The damage-induced autophosphorylation of ATM on S1981 has been demonstrated to promote the retention of ATM to DSBs in a manner that is dependent on the Mre11 complex and the mediator protein, MDC1 (So et al., 2009). Collectively, existing data would support a model that auto and *trans*-phosphorylation could modulate kinase activity, protein–protein interactions, substrate specificity, localization, and chromatin retention of ATM at DSB sites.

### ATM ACTIVATION IN RESPONSE TO CELLULAR STRESS

In addition to DSBs, other types of cellular stress activate ATM in an Mre11 complex-independent manner (Bakkenist and Kastan, 2003; Guo et al., 2010). The ATM INteracting (ATMIN) protein (also known as ASCIZ or ZNF822) was identified as a mediator of ATM activation in response to hypotonic stress or chloroquine treatment (**Figure 2B**; Kanu and Behrens, 2007). ATMIN colocalizes to sites of DNA damage with phosphorylated ATM but deletion of ATMIN does not impair ATM activation or activity following IR treatment. Recent work from Behrens and colleagues has provided compelling evidence that ATMIN and Nbs1 compete for ATM binding as deletion of either protein enhances ATM signaling through the other (Zhang et al., 2012). Strikingly, they showed that the deletion of ATMIN rescued the proliferative defects and premature senescence of Nbs1-deficient cells, suggesting that the loss of Nbs1 resulted in ATMIN-ATM-mediated activation of p53 signaling. Murine cells expressing hypomorphic mutants of Nbs1 that lack the C-terminal ATM interaction domain of Nbs1 (*Nbs1*^Δ C^), a candidate domain for competition with ATMIN, showed normal ATM activation, in conflict with cell line complementation and biochemical data (Falck et al., 2005; You et al., 2005; Difilippantonio et al., 2007; Stracker and Petrini, 2011). One possibility is that ATMIN provides a redundant function in murine cells, where mutant Nbs1 is expressed at physiological levels, that is obscured by the overexpression of mutant forms of Nbs1 in the complementation of human cell lines. A more complete mechanistic understanding of how Nbs1 and ATMIN acquire the attention of ATM and affect its activity will no doubt provide important insights.

### ATM ACTIVATION IN RESPONSE TO REACTIVE OXYGEN SPECIES

Increased ROS has been observed in ATM-deficient tissues and accumulating evidence suggests that this is highly relevant to A-T pathology. Recent work from the Paull lab has demonstrated direct activation of ATM by exposure to ROS *in vitro* (Guo et al., 2010). They proposed that ROS activates ATM by promoting the formation of disulfide bridges involving multiple cysteine residues, including conserved cysteine C2291 in the C-terminal FATC (FRAP, ATM, TRRAP C-terminal) domain (**Figure 2B**). The implications of this mode of activity are very exciting and raise the possibility that this active form of ATM may engage a different set of substrates. Again, how this is coordinated with Mre11 complex or ATMIN-dependent activation in the context of complex cellular stresses will be of great interest and have a potentially high impact on our understanding of ATM regulation and its role in human genetic instability disorders.

## ATM SUBSTRATES AND CELLULAR FUNCTIONS

As ATM is a kinase, its primary role in the DDR is thought to be the phosphorylation of proteins that control signal transduction in response to cellular stresses, such as DSBs and ROS. To date, roughly 1000 proteins have been identified as potential ATM substrates using different approaches (Lavin and Kozlov, 2007; Matsuoka et al., 2007; Mu et al., 2007; Bensimon et al., 2010; Bhatti et al., 2011; Choi et al., 2012). While many bona fide ATM targets have been identified, some of these can be modified by other PIKKs in response to different stress inputs or if ATM is absent. This overlap in substrate specificity has made connecting a particular PIKK to specific targets a formidable challenge in the field. Complicating this is the severe genetic interactions between ATM and DNA-dependent protein kinase catalytic subunit (DNA-PKcs), making the propagation of double mutant cells impossible, as well as the fact that ATR (ATM and Rad3-related) is an essential gene (Brown and Baltimore, 2000; Sekiguchi et al., 2001; Gladdy et al., 2006). Large-scale mass spectrometry in combination with kinase inhibitors has been employed with success and will no doubt be a useful, though imperfect, approach for future studies to identify both direct and indirect targets (Bensimon et al., 2010; Choi et al., 2012). While recent large-scale approaches have broadened the potential roles of ATM in the DDR, and many functions beyond it, its well characterized substrates reflect important regulatory roles in cell cycle progression, DNA repair and the control of cell fate, consistent with the cellular phenotypes of cells lacking ATM.

### ATM AND CELL CYCLE CHECKPOINT REGULATION

A primary role of ATM in the DDR is the activation of cell cycle checkpoints throughout the cell cycle. Defective checkpoint activities were observed in cells from A-T patients over 20 years ago and were speculated to be a major contributor to radiosensitivity (Painter and Young, 1980). Since then, many ATM targets that are critical for checkpoint activation have been identified. These include the tumor suppressor p53, the cohesin subunit SMC1 (structural maintenance of chromosomes proteins), ATF2, the Mre11 complex and additional enzymes involved in the activation of the related ATR kinase and checkpoint kinase 1 (Chk1) through the generation of single-stranded DNA (ssDNA) tails (Shiotani and Zou, 2009; Stracker and Petrini, 2011).

### THE G1/S CHECKPOINT

Cells that experience DNA damage in G1 are prevented from entering S-phase by the G1/S checkpoint that is dependent on the activity of the p53 and has been clearly linked to tumor suppression (Massague, 2004). P53 is one of the first ATM targets to be identified and ATM-deficient tissues and cells show a strong defect in the stabilization of p53 following DNA damage (Siliciano et al., 1997; Banin et al., 1998; Canman et al., 1998). ATM phosphorylates p53 on S15 (S18 in mice) and in conjunction with additional modifications, contributes to p53 stability (Chao et al., 2006). The G1/S checkpoint is impaired in ATM-deficient cells although to a lesser extent than those lacking p53 where the defect is complete (Xu et al., 1998).

Both ATM- and p53-deficient mice are prone to lymphomas that are characterized by complex chromosomal rearrangements in lymphocytes (Zhu et al., 2002; Deriano et al., 2011). As cells undergoing programed rearrangements during V(D)J [variable (V), diversity (D), and the joining (J)] recombination should not enter S-phase until repair takes place, it is likely that defects in this checkpoint play a major role in predisposition to lymphoma (Danska and Guidos, 1997). Consistent with this, mice expressing mutant alleles of p53 that are competent for initiating the G1/S checkpoint have a much longer tumor latency and develop a broader spectrum of non-lymphoma type tumors than p53 alone (Liu et al., 2004; Barboza et al., 2006). While this interpretation is attractive based on known data, the generation of mice expressing a mutant, non-acetylatable form of p53 (p53^3KR^) calls in to question the relationship between DDR signaling mediated by ATM and p53 and tumorigenesis. Mice homozygous for the p53^3KR^ allele showed defective G1/S checkpoint responses as well as impaired apoptosis and senescence in response to DNA damage, but were not prone to rapid tumorigenesis (Li et al., 2012b).

### THE INTRA-S PHASE CHECKPOINT

Cells in S-phase exposed to DNA damage activate the intra-S phase checkpoint, leading to a transient reduction in DNA synthesis and suppression of origin firing. Defects in this checkpoint are characterized by radioresistant DNA synthesis (RDS) that was used as a diagnostic tool and to identify complementation groups in A-T prior to the cloning of the ATM gene (Painter, 1981). Later work showed that RDS was also a feature of cells from NBS and ATLD patients, implicating the Mre11 complex in this checkpoint response (Stracker and Petrini, 2011). The intra-S phase checkpoint is controlled by parallel pathways that are activated by the Mre11 complex and ATM and the checkpoint kinases Chk1 and Chk2 that phosphorylate the CDC25A phosphatase, leading to the inhibition of CDK2 activity and origin firing (Falck et al., 2002). Nbs1, the SMC1 component of cohesin and ATF2 have been identified as a critical ATM targets in the intra-S checkpoint response (Kitagawa et al., 2004; Bhoumik et al., 2005; Difilippantonio et al., 2007; Stracker and Petrini, 2011). However, this checkpoint remains poorly defined at the mechanistic level and the implications of its dysfunction to human health remain unclear, as intra-S defects do not correlate with any severe pathological outcomes in animal models (Kitagawa et al., 2004; Difilippantonio et al., 2007; Stracker and Petrini, 2011; Foster et al., 2012).

### THE G2/M CHECKPOINT

ATM plays a critical role in the activation of the G2/M checkpoint that rapidly prevents G2 cells from entering mitosis after DNA damage. ATM also prevents cells damaged in other phases of the cell cycle from accumulating in G2 at later time points (this is also commonly referred to as the G2/M checkpoint in the literature, although it is mechanistically and genetically distinct; Xu et al., 2001, 2002). Hypomorphic mutations in Mre11 complex alleles, or its depletion by viral proteins, impair ATM activation and the G2/M checkpoint (Williams et al., 2002; Carson et al., 2003; Theunissen et al., 2003). This is also true, although to a lesser extent, in cells with Nbs1 mutations that do not have a pronounced effect on ATM activation (Williams et al., 2002; Uziel et al., 2003). In addition, some mutations in the tumor suppressor BRCA1, an ATM substrate that interacts with the Mre11 complex, impair G2/M checkpoint arrest (Xu et al., 2001, 2002). The deletion of the checkpoint kinase Chk2 in any murine backgrounds with defects in the G2/M checkpoint, including Chk1 heterozygotes, and particular Mre11 complex or BRCA1 alleles, results in tumor predisposition, indicating that the G2/M transition is important for tumor suppression (McPherson et al., 2004; Cao et al., 2006; Stracker et al., 2008; Niida et al., 2010). Understanding how ATM orchestrates the G2/M transition will be important for elucidating its role in disease etiology and will potentially uncover additional candidate proteins involved in oncogenesis (we refer the reader to a recent review of the G2/M transition for more detailed information; Kousholt et al., 2012).

### ATM SIGNALING IN MITOSIS

Available evidence suggests that there is not a general damage-induced checkpoint response in mitosis. However, a basal “priming” response that involves ATM and DNA-PKcs is critical for the normal tolerance of mitotic DNA damage (Giunta et al., 2010). A notable exception is in *Xenopus* where the Costanzo lab identified XCEP63 as a target of ATM/ATR following mitotic DNA damage (Smith et al., 2009). XCEP63 is phosphorylated in an ATM/ATR-dependent manner leading to its detachment from the centrosome and impairment of spindle assembly and mitotic progression. Recently, mutations in human CEP63 were found to underlie autosomal recessive primary microcephaly (Sir et al., 2011). CEP63 was demonstrated to play a key role in the recruitment of CEP152, another centrosomal protein implicated in microcephaly, to centrosomes and the artificial tethering of CEP152 to centrosomes rescued many of the cellular phenotypes of CEP63 mutant cells (Kalay et al., 2011; Sir et al., 2011). Whether the loss of a mitotic DDR, centrosome assembly, or additional functions of CEP63 underlie CNS pathology in humans remains to be determined. The relationship between CEP63 and ATM will require further exploration as the ATM/ATR target site in XCEP63 is not conserved in higher mammals, although additional ATM/ATR consensus SQ/TQ sites have been identified by mass spectrometry.

In addition to regulating the mitotic DDR, ATM has recently been implicated in the regulation of the spindle assembly checkpoint (SAC) through interactions with the Aurora B kinase (Yang et al., 2011). In the absence of DNA damage, ATM was activated in mitosis by Aurora B through the phosphorylation of serine 1403 on ATM. The complementation of ATM-deficient cell cultures with S1403A mutants failed to restore the SAC in contrast to S1981A or wild type ATM expression. While the range of substrates targeted by ATM in mitosis has not been elucidated, the phosphorylation of the Bub1 kinase, a critical regulator of the SAC, by ATM appears to play a major role in ATM-mediated SAC activation. How Aurora B phosphorylation of ATM can initiate its DNA damage-independent activation remains unclear but will be important to understand with regards to the basic mechanisms of ATM activation as well as the role of ATM in promoting chromosome stability.

## REGULATION OF DNA REPAIR AND TRANSCRIPTION IN THE CONTEXT OF CHROMATIN

Although much conflicting data has been reported, recent work has clearly defined multiple roles for ATM activity in regulating both major pathways of DNA repair (NHEJ and HDR) as well as transcription in the vicinity of sites of DNA damage through its activity on numerous chromatin substrates.

### DNA RESECTION COORDINATES THE CELL CYCLE CHECKPOINT AND DNA REPAIR

A key feature of the intra-S and G2 checkpoint responses is the ATM-dependent activation of the ATR and Chk1 kinases through the generation of ssDNA by DNA resection (Shiotani and Zou, 2009). Resection is the conversion of a double-stranded DNA (dsDNA) end to a ssDNA overhang by the nucleolytic removal of one strand in the 5′–3′ direction. The generation of these overhangs is critical for both checkpoint activation and subsequent HDR-mediated repair. Recent work has elucidated a two-step model for DNA-end resection following Mre11 complex end capture (Mimitou and Symington, 2008; Zhu et al., 2008). In the first step, the Mre11 complex and the associated CtIP protein (Sae2/Ctp1 in yeast) initiate limited 5′–3′ resection of the break ends. The identity of the nuclease activity (or more likely, activities) that catalyze this initial step of resection remains an essential question. Recent work from the Neale laboratory provided evidence that in the context of ends with protein blocked termini, Mre11 uses its endonuclease activity to nick the DNA and this is followed by exonucleolytic resection toward the end in a 3′–5′ direction, consistent with the polarity of Mre11 activity (Neale et al., 2005). In concert, exonuclease 1 (Exo1) could act on the same strand in the opposite direction consistent with its 5′–3′ polarity. This elegant bidirectional resection model may be generally applicable to DSB resection, although it is likely that significant redundancy exists, as Mre11 nuclease mutants have mild defects in resection in yeast and a normal G2/M checkpoint in murine cells (Buis et al., 2008; Mimitou and Symington, 2009).

In a second step, additional nucleases, such as Exo1 or DNA2, carry out processive 5′–3′ resection resulting in longer single-stranded tails. These ssDNA tails are bound by replication protein-A (RPA) and serve as a platform to recruit replication factor C (RFC)2–5, the 9–1–1 complex (Rad9, Hus1, Rad1), TOPBP1 and ATRIP that together facilitate the activation of the ATR and Chk1 kinases (Cimprich and Cortez, 2008). In addition to RPA, the single-stranded binding protein complexes SOSS1 and SOSS2 have been implicated in the recruitment of the Mre11 complex, ATM activation and resection via Exo1 (Richard et al., 2008, 2011a,b; Huang et al., 2009; Yang et al., 2012). However, the deletion or depletion of the crucial SOSS1 or SOSS2 components (Obfc2b/SSB1 or Obfc2a/SSB2) in mice does not recapitulate these phenotypes suggesting that they may be context or cell type specific (Feldhahn et al., 2012).

### ATM GOVERNS DNA REPAIR IN HETEROCHROMATIN VIA KAP1

The modulation of chromatin structure is important for DNA repair processes, including resection, particularly in highly condensed regions such as heterochromatin. Recent genome wide studies have demonstrated that mutations in cancer cells occur more frequently in heterochromatin, suggesting that their repair poses a particular challenge to the cell that is relevant to tumorigenesis (Schuster-Bockler and Lehner, 2012). ATM facilitates the relaxation of heterochromatin through phosphorylation of the KAP1 (also known as TIFβ/FRIP1/TRIM28) protein (Ziv et al., 2006; Goodarzi et al., 2008). Depletion of KAP1 by short hairpin RNA (shRNA) rescued the radiosensitivity of lines treated with ATM inhibitor and the complementation of these lines by a non-phosphorylatable KAP1 mutant caused a repair defect, even when ATM was not inhibited. These and other results suggest that the relaxation of chromatin via Kap1 is a major pathway by which ATM regulates radiosensitivity and repair in heterochromatin (the role of ATM and KAP1 in heterochromatin is discussed in detail in a recent review (Goodarzi and Jeggo, 2012)).

### REGULATION OF DSB REPAIR THROUGH RNF20

ATM targets numerous ubiquitin ligases to coordinate transcription and repair following DNA damage. The Shiloh and Komatsu groups both identified the RNF20–RNF40 heterodimer, which normally monoubiquitylates H2B to promote transcription, as an ATM target required for DNA repair through both the HDR and NHEJ pathways (Moyal et al., 2011; Nakamura et al., 2011). The depletion of RNF20 resulted in radiosensitivity and aberrant localization of numerous DDR proteins, including XRCC4, Ku80, and Rad51, which could not be rescued by a non-ubiquitylatable H2B mutant. Monoubiquitylation of H2B is known to occur during transcription where it is followed by the methylation of histone H3K4 and H4K79, the former being required for the recruitment of the SNF2 (sucrose non-fermenting 2) chromatin remodeling protein. While the reports differ in their approaches and assessment of methylation following DNA damage, it was demonstrated using ChIP, under conditions that favored HDR, that H3K4 methylation and SNF2h (sucrose non-fermenting 2 homolog) recruitment occurred at break sites in an RNF20-dependent manner and that the small interfering RNA (siRNA) depletion of RNF20 and SNF2h was epistatic (Nakamura et al., 2011). Collectively, these works have led to the proposition that the ATM-dependent DDR reappropriates the cellular transcriptional apparatus to sites of DNA damage in order to promote chromatin remodeling and facilitate repair through multiple pathways (for a more elaborated perspective on this work we recommend to the reader a recent review from the Shiloh group; Shiloh et al., 2011).

### ATM REGULATES RNAPI- AND RNAPII-DEPENDENT TRANSCRIPTION

Additional links between ATM, the ubiquitylation machinery and transcription was established by Greenberg and colleagues using a novel chromosomal reporter system. They showed that RNA polymerase II (RNAPII)-dependent transcription is silenced in kilobase regions surrounding DSBs (Shanbhag et al., 2010). Transcriptional silencing required ATM activity and was partially dependent upon the H2A ubiquitin ligases RNF8 and RNF168 that have been previously implicated in ATM-mediated chromatin relaxation (Ziv et al., 2006). While reminiscent of previous work that demonstrated that ATM inhibits RNA polymerase I (RNAPI) in a manner dependent upon the Mre11 complex and MDC1, the effects of ATM on RNAPII inhibition appear to be distinct (Kruhlak et al., 2007). The mechanistic details and additional ATM substrates that orchestrate this large-scale regulation of RNAPI and RNAPII, and the consequences of their dysfunction, will be important to determine. Notably, mutations in RNF168 underlie RIDDLE syndrome that has many overlapping pathological features with A-T (Stewart et al., 2009).

## ATM AND THE REGULATION OF CELL FATE IN RESPONSE TO STRESS

Apoptosis, or programed cell death, is essential for development, particularly in the immune system, and represents an important mechanism for the clearance of cells with DNA damage. Apoptosis is triggered in response to a variety of DNA lesions, including DSBs, and defective apoptosis is considered a hallmark of cancer cells (Hanahan and Weinberg, 2011). Apoptosis is also relevant in the context of radio and chemotherapy used for cancer treatment. Cells in the gastrointestinal (GI) tract and the bone marrow undergo extensive cell death in response to DNA damage, potentially causing bone marrow failure and severe GI syndrome. Apoptosis as a response to DSBs is restricted to particular cell types and tissues, as most cell types in the adult do not undergo apoptosis. The exposure to genotoxic stress or the inability to repair persistent DNA damage can also lead to cell death through other mechanisms, such as mitotic catastrophe or necrosis, or the induction of cellular senescence. ATM plays key roles in regulating these cell fate decisions following genotoxic stress that can influence pathological outcomes.

### ATM IN APOPTOSIS

**FIGURE 3:**
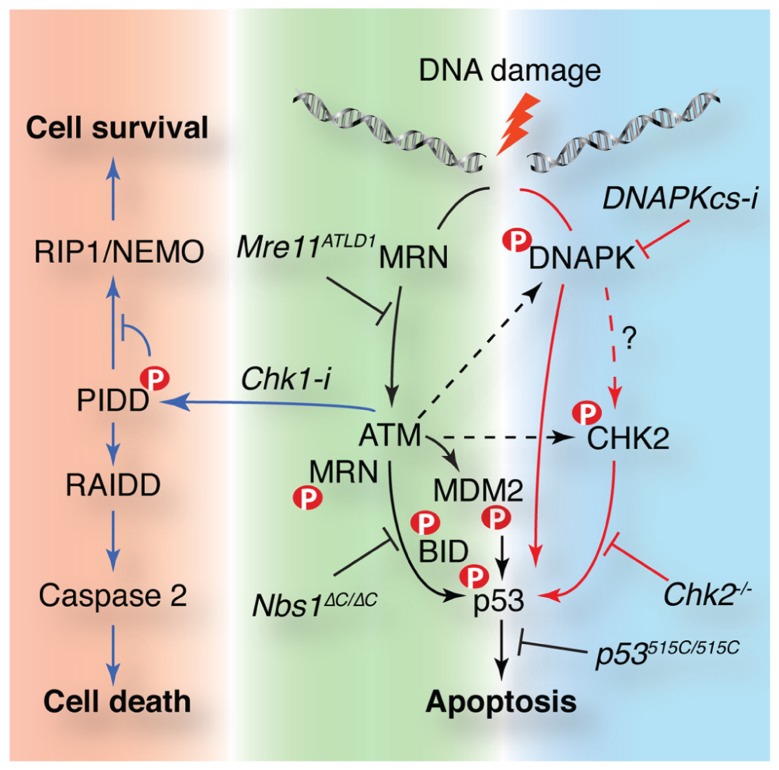
**ATM controls cellular survival in response to DNA damage through multiple pathways.** DNA damage activates apoptosis in a p53-dependent manner in some cell types such as lymphocytes. p53 is activated by parallel pathways controlled by the Mre11 complex and ATM (black arrows) or DNA-PK and Chk2 (red arrows). The Mre11 complex is required for ATM activation and plays downstream roles mediating access to targets such as BID. Chk2 can act independently of ATM and may be a target of DNA-PK that is required for ATM-independent p53-dependent apoptosis. MDM2 phosphorylation by ATM is required for p53 stability and activity. ATM is also required for the Chk1 sensitized cell death pathway. ATM phosphorylates p53-induced protein with a death domain (PIDD) when Chk1 is inhibited favoring RIP associated Ich-1/CED homologous protein with death domain (RAIDD) binding that promotes caspase 2 activation and cell death. Murine alleles or small molecules (Chk1 or DNA-PKcs inhibitors) that affect these pathways are noted in italics. ATM targets are indicated with a red P.

Apoptosis in response to DSBs is regulated by p53 in many tissues, including lymphocytes. Stability of p53 is regulated through its phosphorylation by ATM and Chk2 as well as their regulation of ubiquitin ligases that control p53 levels, namely mouse double minute 2 (MDM2) and murine double minute X (MDMX) (Jenkins et al., 2012). The mutation of both ATM and Chk2 target residues in p53 (p53^S18/23^ mice) to alanine results in apoptotic defects despite the fact that p53 is stabilized to normal levels (Chao et al., 2006). Apoptosis is impaired after DNA damage in both ATM- and Chk2-deficient cells and ATM–Chk2 double mutants have a stronger apoptotic defect, comparable to that of p53 null or p53^S18/23^ animals (**Figure 3**; Stracker et al., 2007; Stracker and Petrini, 2008). Together, these and other data suggested that additional ATM/Chk2 targets besides p53 were required to regulate p53 stability and responses. Recent work from the Jones lab has demonstrated that the phosphorylation of MDM2 by ATM plays a central role in ATM-mediated p53 stabilization and its response to DNA damage (Gannon et al., 2012). Mice expressing a non-phosphorylatable mutant of MDM2 exhibited defects in apoptosis more severe than that seen in ATM deficiency and comparable to that of p53 null animals. This may suggest that this residue can be acted upon by other PIKKs in the absence of ATM. ATM-independent apoptosis in lymphocytes has been shown to require the DNA-PKcs protein using small molecule inhibitors or siRNA depletion (Callen et al., 2009). A possible scenario based on available data is that in the absence of ATM, DNA-PKcs can directly activate Chk2 and modify MDM2 to promote p53-dependent apoptosis (**Figure 3**).

The ability of ATM to activate apoptosis in thymocytes in response to radiation depends largely on the Mre11 complex. Mutations in Mre11 that impair ATM activation mimic the defective apoptosis phenotype of ATM-deficient cells (Stracker et al., 2008). Mutation of the C-terminal ATM interaction domain of Nbs1 also impairs ATM-dependent apoptosis without affecting ATM activation (Difilippantonio et al., 2007; Stracker et al., 2007). Cells lacking the Nbs1 C-terminus show deficient ATM phosphorylation of SMC1 and the proapoptotic BH3 interacting-domain (BID) protein, but normal p53 phosphorylation and stabilization, indicating that this domain controls a subset of ATM targets (Kamer et al., 2005). How the C-terminus of Nbs1 influences ATM-dependent apoptosis remains unclear. As p53 is both phosphorylated (S18) and stabilized properly, it suggests that MDM2 is efficiently phosphorylated and that ATM can access p53 (Gannon et al., 2012). Nbs1 may be required for another critical modification of p53, such as acetylation, or the defect could be kinetic, caused by inefficient regulation of mitochondrial BID or another ATM substrate that is yet to be identified (Li et al., 2012b; Maryanovich et al., 2012).

Recent work has further elucidated the significance of ATM-mediated BID phosphorylation on cell fate and stem cell maintenance in the bone marrow. Previously, ATM has been implicated in regulating the self-renewal of hematopoietic stem cells (HSCs). ATM-deficient mice that escape lymphoma development exhibited progressive bone marrow failure that was rescued by treatment with antioxidants or the inhibition of p38 mitogen-activated protein kinase (p38MAPKs; Ito et al., 2004, 2006). Recent work from the Gross laboratory has identified BID as an ATM substrate and a regulator of HSC homeostasis (Kamer et al., 2005; Zinkel et al., 2005; Maryanovich et al., 2012). Mice expressing a mutant form of BID (BID^AA^) that is not modifiable by ATM were sensitive to radiation and showed impaired bone marrow regeneration. HSCs from these mice were less quiescent, exhibited higher levels of ROS and had increased levels of BID localized to the mitochondria. These results provide exciting links between the ability of ATM to regulate crosstalk between DNA damage, oxidative stress, and the HSC compartment (Maryanovich et al., 2012). It will also be interesting to determine if the Mre11 complex, that is required for efficient IR-induced BID phosphorylation, plays a role in BID-mediated HSC maintenance (Stracker et al., 2007).

As p53 is mutated in a high percentage of cancers, it is of therapeutic interest to identify p53-independent pathways of cell death that can be exploited to improve the efficacy of cancer treatments. Using zebrafish and mammalian cells, the “Chk1 suppressed” (CS) pathway of apoptosis was identified. Inhibition or depletion of Chk1 renders p53-deficient cells competent to induce cell death after DNA damage in an ATM/ATR and caspase 2 (CASP2)-dependent manner (Chen et al., 2009). Recent work from Sidi and colleagues has identified PIDD as an ATM target and critical component of this alternative cell death pathway (Ando et al., 2012). ATM modification of the PIDD death domain promotes its binding to RAIDD, rather than the prosurvival RIP1, to activate CASP2 and cell death (**Figure 3**). The physiological significance of this pathway, as well as the mechanism by which Chk1 prevents its activation, remain unclear but it will be of interest to further delineate these pathways as Chk1 inhibition in p53-deficient tumors is a promising strategy in clinical chemotherapy (Ma et al., 2012; Origanti et al., 2012).

### ATM IN SENESCENCE

In addition to its role in cell death pathways, ATM has also been implicated in the regulation of senescence, although its role remains controversial. ATM-deficient fibroblasts in culture undergo senescence rapidly, likely due in part to the high levels of atmospheric oxygen used in standard culture conditions, as treatment with antioxidants or normoxic conditions prevent premature arrest (Parrinello et al., 2003; Ito et al., 2007; Woodbine et al., 2011). Thus, ATM inhibits senescence in response to oxidative stress. On the other hand, senescence induced by the overexpression of some oncogenes is dependent upon ATM (Bartkova et al., 2006; Mallette et al., 2007; Efeyan et al., 2009). As senescence has emerged as an important mechanism of tumor suppression and aging, it will be critical to clarify the importance of ATM in different contexts (Campisi, 2012). Recent work has elucidated a complex relationship between ATM signaling and the nuclear factor kappa B (NF-κB) and p38MAPK pathways that can regulate cell survival, promote inflammation, and govern cellular senescence in response to DNA damage, ROS, and oncogene expression.

A consequence of senescence is the senescence-associated secretory phenotype (SASP) that can provoke defects in differentiation and promote tumor growth and invasion through the secretion of growth factors and inflammatory cytokines. DNA damage activates a SASP in primary fibroblasts that is dependent upon ATM, Nbs1, and Chk2 (Rodier et al., 2009). ATM regulates a subset of the SASP including IL-6 and IL-8. The Campisi group has demonstrated that the inhibition of the p38MAPK pathway blocks the SASP following DNA damage independently of ATM and Nbs1 (Freund et al., 2011). Activity of p38MAPK activity is observed later than the initial DDR and is restrained by p53, suggesting that an attenuation of ATM and Chk2 pathways may be a prerequisite. Overexpression of a constitutively active p38MAPK activator MKK6 (MKK6EE) activated a SASP, demonstrating that high levels of active p38MAPK was sufficient. However, DNA damage induction was also required for optimal induction with several experimental systems that do not induce p38MAPK activation as strongly. The activation of the NF-κB pathway was required for SASP induction downstream of p38MAPK and the authors have proposed that the DDR and p38MAPKs may co-regulate NF-κB post-translationally to regulate the expression of its target genes and the SASP.

The NF-κB pathway is activated in an ATM-dependent manner in response to some types of DNA damage (Wu et al., 2006). ATM phosphorylates the NF-κB essential modulator (NEMO), leading to its ubiquitylation and the export of NEMO and ATM to the cytoplasm. NF-κB signaling by ATM has been linked to immune system development, DNA repair, tumor progression, and defects in the nuclear lamina (Bredemeyer et al., 2008; Barascu et al., 2012; McCool and Miyamoto, 2012; Osorio et al., 2012). Work from the Lopez-Otin group recently described NF-κB activation in an ATM- and NEMO-dependent manner in mice lacking the metalloproteinase Zmpste24 that processes prelamin A to lamin A (Osorio et al., 2012). This response underlies the progeroid features of mice lacking Zmpste24 that can be partially rescued by decreasing the gene dosage of lamin A.

Additional links between ATM and the nuclear lamina were uncovered by the Bertrand laboratory that found lamin B protein, but not mRNA, is overexpressed in ATM-deficient cells, leading to morphological defects (Barascu et al., 2012). Lamin B stabilization was dependent upon oxidative stress and a functional p38MAPK pathway. The mechanism by which ROS and p38MAPK promote lamin B stabilization remains unclear but impaired proteasome-mediated protein degradation caused by elevated expression of an ubiquitin-like protein ISG15 in the brain of A-T patients, as well as in ATM-deficient mice has been reported (Wood et al., 2011). ISG15 counteracts Ub-dependent proteasome degradation, and thus could provide a mechanistic explanation for the stabilization of proteins such as lamin B, as it targets many cytoskeletal proteins including lamin A (Zhao et al., 2005). How defects in the integrity of the nuclear lamina activate ATM remains unclear but as the lamin B receptor (LBR) has been linked to heterochromatin organization in the nucleus, and lamin B loss is a biomarker of senescence, understanding these connections will no doubt be informative (Goldberg et al., 1999; Freund et al., 2012).

### ATM AND ONCOGENE-INDUCED STRESS

The overexpression of some oncogenes, such as c-Myc, can cause replicative stress, leading to an active DDR and the initiation of apoptosis or senescence depending on the cellular background. In the case of c-Myc, ATM and p53 have been identified as central mediators of c-Myc signaling (Campaner and Amati, 2012). Loss of ATM accelerates c-Myc-induced tumorigenesis in both an epithelial tumor (K5-myc) and lymphoma model (Eμ-myc) in part by reducing apoptosis and augmenting proliferation (Pusapati et al., 2006; Maclean et al., 2007). Although it was not assayed as an endpoint in many previous studies, separation of function mutations in p53 implicate senescence as a major barrier to c-Myc-induced tumorigenesis in lymphocytes (Post et al., 2010). Consistent with this, deletion of the WIP1 phosphatase that restrains ATM and p53 activity, as well as both the NF-κB and p38MAPK pathways, delays Eμ-myc-induced tumorigenesis in a manner that requires both p53 and ATM (Bulavin et al., 2004; Shreeram et al., 2006a,b; Demidov et al., 2007; Chew et al., 2009). In contrast to the augmentation of c-Myc driven tumorigenesis in mice lacking ATM or p53, the mutation or inhibition of ATR leads to the converse outcome of impaired tumor development (Murga et al., 2011). These results highlight the inherent differences in the cellular roles of the ATM and ATR signaling pathways and have suggested that ATR pathway inhibition has potential as a chemotherapeutic strategy, particularly in oncogene addicted tumors (Toledo et al., 2011; Schoppy et al., 2012).

The influence of ATM deficiency on c-Myc-induced tumorigenesis is no doubt complex and involves multiple ATM targets. One target that may be of particular interest is the ubiquitin-specific protease 28 (USP28) that was identified as an ATM/ATR target through its interactions with 53BP1 and independently through a screen for genes required for Myc-induced transformation (Zhang et al., 2006; Popov et al., 2007b). USP28 has also been implicated in Chk2-dependent apoptosis as well as the maintenance of the ATM-dependent G2 checkpoint through the ability to stabilize the claspin protein (Bassermann et al., 2008). Based on these and other data, it has been proposed that DDR-induced phosphorylation of USP28 leads to its dissociation from the Fbw7 ubiquitin ligase complex, allowing unchecked Myc degradation, and its subsequent association with DDR components, such as 53BP1, claspin, Nbs1, and Chk2, to promote their stabilization (Popov et al., 2007a). This model predicts that mice or human patients lacking USP28 would fail to coordinate Myc levels with DNA damage signaling, potentially causing replication stress and enhanced genomic instability that could contribute to cancer (Shah et al., 2009).

## DIVERSE ROLES OF ATM IN IMMUNITY

Ataxia-telangiectasia patients are more susceptible to infections and this has been attributed in part to varying degrees of immunodeficiency. Work in mice- and cell-based systems has identified a number of important roles for ATM in both adaptive and innate immunity, as well as inflammatory responses, that may underlie many aspects of pathology in A-T.

### ADAPTIVE IMMUNITY

ATM plays an important role in the development of both T and B cells and A-T patients often exhibit abnormal T and B lymphocyte counts and deficient antibody responses. Lymphocytes are the main cellular component of the adaptive immune system that counteracts infections and cell abnormalities, including cancer. To react to a wide range of antigens, lymphocytes generate a diverse repertoire of antigen-specific receptors that depend on programed chromosomal rearrangements that are initiated by enzymes that introduce DNA breaks. These processes, V(D)J recombination (T and B lymphocytes) and class switch recombination (CSR, in B lymphocytes), are both dependent on intact ATM function. As A-T patients are prone to lymphomas, it is likely that the ability of ATM to monitor the development of lymphocytes through the regulation of both DNA repair and apoptosis plays a critical role in tumor suppression (Nussenzweig and Nussenzweig, 2010).

The lymphoid organs of mice lacking ATM are structurally intact but the absolute numbers of thymocytes are reduced due to developmental defects. The thymocyte population in ATM null mice is characterized by a reduction in mature single positive CD4 or CD8 T cells and an increase in immature double positive thymocytes (Barlow et al., 1996; Xu et al., 1996). Normal numbers of B cells are present in the spleen but a reduction in the number of B220^+^IgM^-^ Pre-B cells was observed in the bone marrow. Moreover, B cells are unable to respond properly to stimuli due to defects in B cell homeostasis and the regulation of programed rearrangements during V(D)J and CSR. Recent work supporting this has demonstrated that ATM-deficient animals are able to stimulate T and B cell responses in response to chronic gammaherpesvirus infection, but that these responses are defective and unable to efficiently suppress viral replication (Kulinski et al., 2012).

### V(D)J RECOMBINATION

During the development of T and B lymphocytes, V(D)J recombination is required for the assembly of antigen receptor genes. The recombination activating genes 1 and 2 (RAG1 and RAG2) constitute the RAG recombinase that generates DNA DSBs to catalyze recombination between the variable (V), diversity (D), and the joining (J) gene fragments in order to define the binding properties of the receptor (Boboila et al., 2012). NHEJ, one of the two major DSB repair pathways, is critical for the repair of RAG-induced breaks. The so-called “classical” or C-NHEJ pathway is defined as the DNA-PK holoenzyme (Ku70/80, DNA-PKcs), XRCC4, Lig4, Artemis, and XLF. The C-NHEJ pathway repairs the hairpin capped coding ends (CEs) and the blunt signal ends (SEs) generated by RAG activity. The kinase activity of DNA-PKcs stimulates the activity of the Artemis endonuclease that is required for the repair of CEs while the remaining C-NHEJ factors are critical for repair of both CE and SE ends. Blunt SE ends are repaired to a large extent in cells lacking DNA-PKcs or Artemis but not completely to normal levels (Boboila et al., 2012).

ATM colocalizes with RAG at endogenous recombination loci and RAG-induced DNA breaks persist in ATM-deficient cells due to incomplete defects in CE repair (Perkins et al., 2002; Callen et al., 2007; Nakamura et al., 2011). Persistent breaks in the immunoglobulin heavy chain (IgH) locus promote translocations, including those with Myc, that are known to promote lymphomagenesis and occur at higher rates in ATM-deficient cells (Ramiro et al., 2006). Recent work from the Alt lab has revealed an unexpected redundancy between ATM and other end-joining factors in the repair of both CE and SEs. Cells lacking both DNA-PKcs and ATM activity show a strong reduction in the level of SE repair (Zha et al., 2011b) and mice lacking both XLF and ATM exhibit reduced SE and CE joining (Zha et al., 2011a). Collectively, these and other studies suggest that ATM plays a role in monitoring C-NHEJ-mediated repair during V(D)J recombination. This likely occurs through multiple activities that include the promotion of RAG complex stability, the activation of checkpoints to prevent the propagation of persistent breaks, the control of DNA end usage and the regulation of target proteins, such as H2AX and Artemis, that are shared with DNA-PKcs (Danska and Guidos, 1997; Bredemeyer et al., 2006; Callen et al., 2007; Bennardo and Stark, 2010; Deriano et al., 2011; Zha et al., 2011a).

### CLASS SWITCH RECOMBINATION

In response to cytokine secretion or infections, B cells initiate antibody class switching (Boboila et al., 2012). This stimulation leads to rearrangements of the switch regions, thus altering the effector function of the antibody. Class switching is catalyzed by the activation-induced deaminase (AID) that promotes strand breakage. Human A-T patients frequently have impaired development of the immunoglobulin subtypes IgA, IgG2, IgG4, and IgE in the serum and ATM-deficient mice show strong defects in CSR (Lahdesmaki et al., 2000; Pan et al., 2002; Lumsden et al., 2004; Reina-San-Martin et al., 2004). In contrast, AID-dependent somatic hypermutation is not strongly affected by ATM deficiency (Pan-Hammarstrom et al., 2003; Lumsden et al., 2004).

Class switch recombination depends on the core C-NHEJ machinery but also has distinct requirements from V(D)J. The mechanism by which ATM regulates CSR is likely complex and represents a composite of the misregulation of many substrates. The ATM substrate 53BP1 is critical for both V(D)J and CSR where it has been proposed to play multiple roles including the synapsis of distal ends and the protection of free ends from DNA resection (Difilippantonio et al., 2008; Dimitrova et al., 2008; Bothmer et al., 2010; Bunting et al., 2010). The localization of 53BP1 to DNA breaks is dependent on H4K20 methylation and the PIKK-dependent phosphorylation of H2AX (Celeste et al., 2003; Bothmer et al., 2011). 53BP1 is phosphorylated on 28 consensus PIKK SQ/TQ target sites and the mutation of all of these residues to alanine impairs the ability of 53BP1 to support CSR and block resection, but does not prevent its localization to γH2AX containing breaks (Bothmer et al., 2011).

Recent work from the de Lange and Nussenzweig laboratories has shed light on the relevance of 53BP1 phosphorylation by ATM as they have demonstrated the PIKK consensus sites are required for its interactions with the RIF1 protein and the regulation of DNA resection (Jankovic et al., 2013; Zimmermann et al., 2013). In lymphocytes, the interaction between 53BP1 and RIF1 is ATM-dependent and the deletion of RIF1 leads to increased AID-dependent breaks in the IgH locus, an accumulation of cells in G2/M and extensive 5′–3′ resection. The Casellas and Nussenzweig labs have linked the aberrant resection of AID-induced breaks in the absence of 53BP1 to lymphomagenesis using an innovative high-throughput sequencing approach that mapped AID-induced translocations as well as the asymmetric binding of the ssDNA binding protein RPA, allowing them to monitor resection (Jankovic et al., 2013). Further work will be required to understand precisely how ATM-deficient cells bypass the severe phenotypes of RIF1 and 53BP1 mutants in CSR, potentially through the redundant functions of other PIKK activities such as DNA-PKcs.

## ATM IN FERTILITY

Due to the young age of A-T patients, fertility is often overlooked as a clinical issue. However, ATM plays critical roles in germline development as A-T patients present with gonadal dysgenesis and both male and female mice lacking ATM are infertile due to defects in meiotic progression (Barlow et al., 1996). Meiosis is the special cell division that ensures the formation of haploid cells, spermatozoa and ova, from diploid progenitor germ cells. Germ cells undergo two rounds of chromosome segregations after replicating their genomes only once. During the first meiotic division, DSBs are generated by the SPO11 protein and repaired by homologous recombination (HR; Keeney et al., 1997). HR leads to the synapsis of homologous chromosomes that are stabilized by the synaptonemal complex (SC), formed by the union of the two chromosomal core axes by a central element. Repair of DSBs in meiosis can lead to the formation of non-crossovers (NCO, when a small region of the homologous chromosome is used as template to repair the damage) or crossovers (CO, when flanking regions around the DSBs are exchanged between the homologs). The formation of the right number of COs is critical to properly segregate the homologs in the first meiotic division and reduce the genome by half. In fact, inaccurate CO distribution in human oocytes is believed to be the major cause of human aneuploidy (Nagaoka et al., 2012).

ATM has a crucial role in the completion of mouse gametogenesis since both male and female ATM-deficient mice are sterile (Barlow et al., 1996). Testes and ovaries from *ATM* null animals display massive germ cell loss. While *ATM*^-/-^ spermatocytes arrest at the pachytene stage of meiotic prophase, *ATM*^-/-^ females lose all oocytes during the first days of life, before completing meiotic prophase (Barlow et al., 1998; Hamer et al., 2004; Di Giacomo et al., 2005). The cytological analysis of *ATM*^-/-^ spermatocytes revealed that ATM is required to complete homologous chromosome synapsis (Barlow et al., 1998; Pandita et al., 1999). SCs in ATM mutant spermatocytes are fragmented and this is dependent on DSB formation, as *Spo11*^-/-^ ATM^-/-^ spermatocytes do not show broken SCs (Bellani et al., 2005). Moreover, the ends of the fragmented axes from *ATM*^-/-^ spermatocytes contain recombination markers such as γH2AX, RPA, or RAD51 (Plug et al., 1997; Barchi et al., 2008).

As expected from the presence of these synaptic defects, recombination is compromised in *ATM*^-/-^ spermatocytes. H2AX phosphorylation, that arises as a response to programed DSB formation in early meiotic prophase, is delayed, implying that ATM is involved in the early steps of meiotic recombination (Fernandez-Capetillo et al., 2003; Barchi et al., 2005; Turner et al., 2005). Accordingly, the assembly of RAD51 foci is inefficient and mislocalized in *ATM*^-/-^ spermatocytes. While RAD51 foci colocalize with the SC in wild type cells, multiple foci also form in the chromatin of *ATM* mutant cells (Barlow et al., 1997, 1998).

The gonadal pathology of *ATM*^-/-^ mice is strongly influenced by the failure to complete meiotic recombination as it is partially rescued by the heterozygosity of *Spo11* that reduces DSB formation to 70–85% of wild type levels (Bellani et al., 2005; Barchi et al., 2008; Cole et al., 2012). In this background, *ATM*^-/-^ spermatocytes are able to complete meiotic prophase. Nevertheless, *Spo11*^+/-^ ATM^-/-^ spermatocytes are unable to complete meiotic recombination because markers of unrepaired breaks, like phosphorylated H2AX, can be found even in cells that have completed meiotic prophase (Barchi et al., 2008). The persistence of DSBs or recombination intermediates normally provokes a checkpoint response that delays or stops meiotic progression, and can in some cases initiate programed cell death (MacQueen and Hochwagen, 2011). In mice, DSB-dependent arrest in meiosis is believed to occur at the pachytene stage of meiotic prophase I (de Rooij and de Boer, 2003). However, despite exhibiting multiple unrepaired DSBs, *Spo11*^+/-^ ATM^-/-^ spermatocytes do not arrest at pachynema and progress to metaphase I (Bellani et al., 2005; Barchi et al., 2008). This uncoupling of DSB repair and meiotic prophase progression suggests that ATM could be required as part of a checkpoint mechanism that controls meiotic development in the mouse, a possibility that remains to be formally tested.

Heterozygosity of *Spo11* has also revealed that ATM is involved in CO control, as *Spo11*^+/-^ ATM^-/-^ cells have more COs than wild type cells (Barchi et al., 2008). Interestingly, *Spo11*^+/-^ ATM^-/-^ spermatocytes fail to form the obligate CO between the X and Y chromosomes, that contain a small region of homology that guarantees their proper segregation at the end of the first meiotic division. Furthermore, COs are more closely positioned in *Spo11*^+/-^ ATM^-/-^ mutants than in *Spo11*^+/-^ spermatocytes, suggesting that ATM not only is involved in controlling the number of COs formed, but also in their proper position and spacing, both crucial aspects for allowing proper homologous chromosome segregation. These studies reveal that ATM may have an important role in coordinating meiotic chromosome dynamics, including chromosome axis formation as well as recombination (Barchi et al., 2008). Thus, the tight relationship between SC defects and inefficient recombination in ATM-deficient cells suggests that ATM could coordinate these two mechanisms to ensure proper meiotic prophase progression in mouse spermatocytes.

Interestingly, mice with hypomorphic alleles of the *Mre11* or *Nbs1* genes exhibited meiotic defects similar to that of *Spo11*^+/-^ ATM^-/-^ mice. *Mre11*^ATLD1/ATLD1^ or *Nbs1*^ΔB/ΔB^ spermatocytes showed aberrant synapsis among homologous chromosomes, persistence of recombination markers until late meiotic prophase and altered number and location of COs (Cherry et al., 2007). These findings suggest that the Mre11 complex participates in meiotic recombination but more importantly, that most ATM functions during meiosis may be dependent on the Mre11 complex. Nevertheless, it is worth noting that, unlike *Spo11*^+/-^ ATM^-/-^ mice, both *Mre11*^ATLD1/ATLD1^ and *Nbs1*^Δ B/Δ B^ male mice are subfertile. This may imply that some of the meiotic activities of ATM are independent of the MRN complex function or that leaky ATM function in these Mre11 complex mutant backgrounds is sufficient for some degree of fertility (Theunissen et al., 2003).

Recently, the Jasin and Keeney labs reported that ATM is involved in controlling the number of meiotic DSBs created by Spo11 (Lange et al., 2011). Detecting the DNA oligonucleotides covalently linked to Spo11 as a readout of DSB formation, they showed that *ATM*^-/-^ mice have up to 10-fold more DSBs than wild type littermates (Neale et al., 2005; Lange et al., 2011). This was not due to differences in the cellularity of the mutant testis, as other mutants that arrest at the same stage as *ATM*^-/-^, such as *Dmc1*^-/-^, have approximately a 50% reduction in the amount of Spo11-oligo complexes compared to wild type testis. They also showed that DSB formation was dependent on Spo11 expression since *Spo11*^+/-^ ATM^-/-^ had fewer DSBs than *ATM*^-/-^ spermatocytes, but still significantly more than wild type cells. Moreover, cells expressing two extra copies of the Spo11β in an *ATM*^-/-^ background had even more DSBs than *ATM*^-/-^ spermatocytes, something that was not observed in an ATM proficient background. This work highlights the remarkable control of DSB formation in meiotic cells, revealing that it may be similarly deleterious for a meiocyte to form too few or too many DSBs. The authors propose that activation of ATM by DSB formation would create a local negative feedback loop that would inhibit Spo11 activity in the vicinity of a DSB. This mechanism might be important to evenly space DSBs along the genome, in order to promote proper homologous synapsis, as well as minimizing the formation of DSBs in both sister chromatids in the same region of the genome, which could impair meiotic recombination.

## ATM IN THE DEVELOPMENT OF THE CENTRAL NERVOUS SYSTEM

For reasons that remain largely unclear, brain development is highly susceptible to defects in the DDR. Unlike the immune system or germline, there are no known programs of DNA breakage and repair that would provide an obvious trigger for cell death. It has been speculated that mitochondrial defects, the accumulation of ROS, transposon mobilization, innate immune responses, the regulation of apoptosis or specific repair pathway defects may contribute to triggering neuronal cell death (Coufal et al., 2011; McKinnon, 2012; Petersen et al., 2012; Valentin-Vega and Kastan, 2012). One of the central pathologies associated with A-T is neurodegeneration, characterized by cerebellar atrophy and the loss of Purkinje and granule cells and subsequent ataxia. Notably, human diseases with the most similar neuropathology to A-T are caused by genes involved in the repair of diverse types DNA lesions (for more information on this topic, as well as the general neuropathology of A-T, we recommend to the reader this recent review; McKinnon, 2012). Unfortunately, neurodegeneration remains the most poorly understood aspect of A-T, as pronounced neurodegeneration and ataxia are not observed in mice lacking ATM. However, many insights have been gleaned from animal models and other systems that provide insight into the roles of ATM and the DDR in the CNS.

Central nervous system pathology is a feature common to many diseases caused by mutations in genes that encode members of the ATM-dependent DDR, including *MRE11* (ATLD), *NBS1/NBN* (NBS), *RAD50* (NBSLD), *RBBP8/CtIP* (Seckel), *ATR* (Seckel), *CEP63* (MCPH), *PNKP* (MCSZ), *TDP1* (SCAN1), and *ATM* (A-T; Das et al., 2009; Waltes et al., 2009; Segal-Raz et al., 2011; Sir et al., 2011; Stracker and Petrini, 2011; McKinnon, 2012; Poulton et al., 2012; Reynolds et al., 2012). However, depending on the particular mutation, patients, and in some cases, animal models, will develop either microcephaly (defined as a head circumference two-standard deviations smaller than the average) or neurodegeneration (defined as the progressive loss of neurons). It remains mechanistically unclear why mutations in *MRE11* result in neurodegeneration, similar to what is observed in A-T patients, while other mutations in *NBS1* or *RAD50* cause microcephaly.

Characterization of hypomorphic *Nbs1* and *Mre11* alleles in mice has revealed differential influences on the ATM-dependent DDR. The *Mre11*^ATLD1^ allele impairs ATM activation, thus reducing the total pool of active ATM and potentially affecting all substrates (Theunissen et al., 2003). In contrast, the *Nbs1*^Δ B^ allele does not impair ATM activation but reduces ATM activity on particular substrates (e.g., SMC1), while not affecting others (e.g., p53; Stracker et al., 2008). One clear phenotypic difference lies in the ability of these alleles to induce apoptosis following IR treatment (Stracker et al., 2007, 2008). Activation of p53-dependent apoptosis is impaired in the thymus and GI tract of *Mre11*^ATLD1^ animals but is indistinguishable from wild type in *Nbs1*^Δ B^. Using both IR treatment and Lig4 deficiency to induce apoptosis in the developing brain, it has been demonstrated that this difference in apoptosis proficiency also applies to the CNS (Shull et al., 2009). An attractive possibility proposed from this work is that the status of apoptosis may dictate whether there is cellular attrition during development, leading to microcephaly, or whether cells experiencing genotoxic stress survive and are lost later due to other cell death pathways triggered by genomic instability, causing neurodegeneration. More recently, ATLD patients with microcephaly, rather than neurodegeneration, have been identified as well as NBSLD patients with microcephaly due to *RAD50* mutations (Waltes et al., 2009; Matsumoto et al., 2011). Determining if these *MRE11* or *RAD50* alleles impair apoptosis would provide a means for testing this proposition. The major question remains as to what triggers the DDR in the CNS and whether a few common or many diverse mechanisms are at play in the human diseases.

ATM deficiency is synthetically lethal with both the *Mre11*^ATLD1^ and *Nbs1*^Δ B^ alleles, hampering efforts to identify independent effects or potential redundancies in development (Williams et al., 2002; Theunissen et al., 2003). Deletion of any of the Mre11 complex members, including Nbs1, is embryonic lethal. Nevertheless, using conditional alleles, it has been demonstrated that CNS-specific loss of Nbs1 leads to microcephaly, defects in the development of the cerebellum and ataxia, in contrast to ATM-deficient mice or hypomorphic Nbs1 alleles that do not exhibit ataxia (Kang et al., 2002; Williams et al., 2002; Frappart et al., 2005; Stracker and Petrini, 2011). Many of these defects were rescued by loss of p53 and p53 activation was observed in CNS tissues where Nbs1 was deleted, suggesting that ATM activation may play a role in the pathological outcomes resulting from Nbs1 deficiency (Frappart et al., 2005). However, in contrast to p53 deficiency, loss of ATM exacerbated the microcephaly and ataxia phenotypes of CNS-specific Nbs1 deletion, again confirming that ATM and the MRN complex make independent contributions to CNS development (Dar et al., 2011). In addition, ATM-deficient mice with a deletion of Nbs1 in the CNS showed impaired growth and a markedly shortened life span. It would be interesting to compare the CNS of mice lacking both Nbs1 and ATMIN as ATMIN deletion elicits neurodegeneration and rescues other cellular phenotypes of Nbs1 null tissues (Kanu et al., 2010; Zhang et al., 2012). Whether ATMIN deletion would rescue CNS development by impairing ATM-mediated p53 activation or enhance the defects, as seen in mice lacking ATM, would be mechanistically informative.

One explanation invoked for the synthetic lethality of Mre11 complex mutations and ATM deficiency is the role of the Mre11 complex in activating ATR (Williams et al., 2002). Loss of ATR is embryonic lethal and hypomorphic mutations mimicking those found in human Seckel syndrome result in severe microcephaly in mice and are synthetically lethal with ATM deficiency (Brown and Baltimore, 2000; Murga et al., 2009; Ragland et al., 2009). The conditional deletion of ATR in the CNS also causes microcephaly and defective cerebellar development (Lee et al., 2012). However, in contrast to the deletion of Nbs1 in the CNS, the resulting pathology was not dependent on p53 (Murga et al., 2009). The balance of ATM and ATR signaling in the development of the CNS, and the organism as a whole, is clearly of great importance but remains poorly understood. Cleanly separating their functions and accounting for the potential redundancy of DNA-PKcs signaling remains an important challenge to overcome.

Recent work from the Herrup lab has linked ATM’s role in CNS development to transcriptional regulation in neurons. They proposed that transcriptional defects caused by the aberrant nuclear localization of HDAC4 in ATM-deficient cells may contribute to neurodegeneration (Li et al., 2012a). Using a variety of approaches including ChIPseq, they showed that HDAC4 accumulated in the nucleus in ATM-deficient neurons and caused global defects in histone acetylation and neuronal gene expression. Of particular interest, nuclear HDAC4 suppressed the activity of myocyte enhancer factor 2A (MEF2A) and cAMP-responsive element binding protein (CREB) that control prosurvival programs. Treatment with histone deacetylase inhibitors reduced cell death and markers of cell cycle reentry in the ATM-deficient cerebellum. HDAC4 localization does not appear to be regulated directly by ATM-mediated phosphorylation but instead through ATM activity on the P65 subunit of PP2A, a known interactor of ATM (Goodarzi et al., 2004). ATM phosphorylation of PP2A promoted its cytoplasmic localization and prevented the dephosphorylation of HDAC4. The injection of mutant HDAC4 that localized only to the cytoplasm, coupled with shRNA downregulation of endogenous HDAC4, rescued some of the behavioral defects of ATM-deficient mice and reduced markers of cell death, providing a proof of principle for HDAC4 modulation in therapy. HDAC4 is expressed in many areas of the brain as well as other tissues, thus the pathological specificity of ATM loss on neurons remains unclear. It will be important to understand as well how HDAC4 is regulated in similar diseases resulting from Mre11 complex mutations to understand if this mechanism is particular to A-T or may have more widespread significance in genetic instability diseases.

## THE ROLES OF ATM IN METABOLISM

Ataxia-telangiectasia patients exhibit several indices of metabolic disease including increased susceptibility to diabetes and impaired glucose metabolism. Some early indications for molecular roles of ATM in metabolism came from the identification of the translation regulator 4E-BP1 as an ATM target in response to insulin, the observation that ROS is increased in ATM-deficient animals and evidence for the peroxisomal localization of ATM (Barlow et al., 1999; Watters et al., 1999; Yang and Kastan, 2000; Kamsler et al., 2001). Subsequently it has been demonstrated that treatment with antioxidants could delay tumor formation in ATM null mice and rescue other aspects of development (Ito et al., 2004, 2007; Schubert et al., 2004; Reliene and Schiestl, 2006, 2007; Reliene et al., 2008). The recent demonstration that ROS can directly activate ATM, that ATM can promote antioxidant responses through the stimulation of the pentose phosphate pathway (PPP) and evidence that ATM plays a role in monitoring mitochondrial quality control, has again pointed toward a central function of ATM in controlling cellular ROS metabolism (Guo et al., 2010; Cosentino et al., 2011; Valentin-Vega et al., 2012; these topics will be covered in brief here but we refer the readers to recent reviews/commentaries that cover some aspects in greater depth; Alexander et al., 2010b; Ditch and Paull, 2012; Valentin-Vega and Kastan, 2012).

Autophagy, the catabolism of dysfunctional or excess cellular components, is induced in response to diverse stresses including elevated ROS. In response to ROS, cytoplasmic ATM phosphorylated liver kinase B1 (LKB1) and activated AMP-activated protein kinase (AMPK; Alexander et al., 2010a). Together LKB1 and AMPK activated TSC2 that in turn repressed the mammalian target of rapamycin complex 1 (mTORC1) and induced autophagy. Recent work from the Kastan lab has shown that ATM-deficient thymocytes exhibited increased ROS and mitochondrial mass, as well as other markers of mitochondrial dysfunction (Valentin-Vega et al., 2012). Cells from ATM mutant animals have increased basal autophagy but defects in mitophagy induced by mitochondrial membrane decoupling agents. A reduction in autophagy, by the deletion of one allele of the Beclin1 gene, reverted some of the mitochondrial phenotypes and significantly delayed tumor suppression without affecting the DDR, providing evidence that autophagy promotes tumorigenesis in the absence of ATM. In addition, ATM localized to mitochondrial fractions and was activated in response to mitochondrial membrane uncoupling. These and other data have led to the proposal that ATM may directly regulate mitochondrial homeostasis through responding to ROS or by regulating mitochondrial quality control genes, such as PINK1 or Parkin (Valentin-Vega and Kastan, 2012). As these proteins are mutated in Parkinson’s disease, also characterized by CNS degeneration, understanding the regulation of mitochondrial integrity by ATM may shed light on the etiology of neurodegeneration in A-T.

Mitochondrial dysfunction can lead to excessive ROS generation that is a phenotype of ATM-deficient cells that has been described in many experimental settings, including those mentioned here in previous sections. Recent work from the Costanzo lab has defined a role for ATM in activating the PPP in response to DNA damage (Cosentino et al., 2011). ATM increased the activity of glucose-6-phosphate dehydrogenase (G6PD) by promoting its interaction with heat shock protein 27 (HSP27). This led to an increase in the production of nicotinamide adenine dinucleotide phosphate (NADPH), an essential co-factor for antioxidants and ribonucleotide reductase, as well as ribose-5-phosphate, that can be used for nucleotide biosynthesis. Interestingly, HSP27 is an important downstream target of the p38MAPK pathway and inhibition of p38 activity impaired the ATM-mediated stimulation of G6PD and phosphorylation of HSP27 on a target site regulated by p38MAPK (Cosentino et al., 2011). This indicated that p38MAPK activity was stimulated by ATM and required for ATM to promote an antioxidant defense through the PPP. In contrast, several reports have implicated p38MAPK activity in phenotypic outcomes resulting from increased ROS in the absence of ATM, including lamin B accumulation, impaired HSC homeostasis, impaired neural stem cell proliferation, cytokine secretion, and defective neoangiogenesis (Kim and Wong, 2009; Freund et al., 2011; Barascu et al., 2012; Maryanovich et al., 2012; Okuno et al., 2012). Understanding the complex relationship between ATM and p38MAPK signaling pathways will no doubt be of central importance in elucidating the mechanisms by which ATM modulates metabolism and suppresses a wide range of pathological outcomes.

Mice expressing hypomorphic Mre11 complex alleles recapitulate most DDR related aspects of ATM deficiency but are not tumor prone and do not exhibit the same oxygen-dependent phenotypes observed in the absence of ATM (Williams et al., 2002; Theunissen et al., 2003; Ito et al., 2004; Difilippantonio et al., 2005; Stracker et al., 2007; Valentin-Vega et al., 2012). It is therefore likely, at least in mice, that ROS signaling via ATM is largely intact and this could be sufficient for tumor suppression in Mre11 complex mutants. However, the clinical presentation in human patients of the same *Mre11*^ATLD1^ mutation modeled in mice is very similar to that of A-T, arguing against this possibility (Stewart et al., 1999; Theunissen et al., 2003). As so few ATLD patients with any one *MRE11* allele have been identified, it is difficult to definitively compare tumor predisposition, but as mitochondrial dysfunction and the response to ROS has been implicated as a major influence on the disease phenotypes, including neurodegeneration and tumorigenesis, it will be important to understand this aspect of ATM signaling and to what extent it is influenced or not by the Mre11 complex, ATMIN or other regulators or substrates of ATM.

## CONCLUDING REMARKS

**FIGURE 4:**
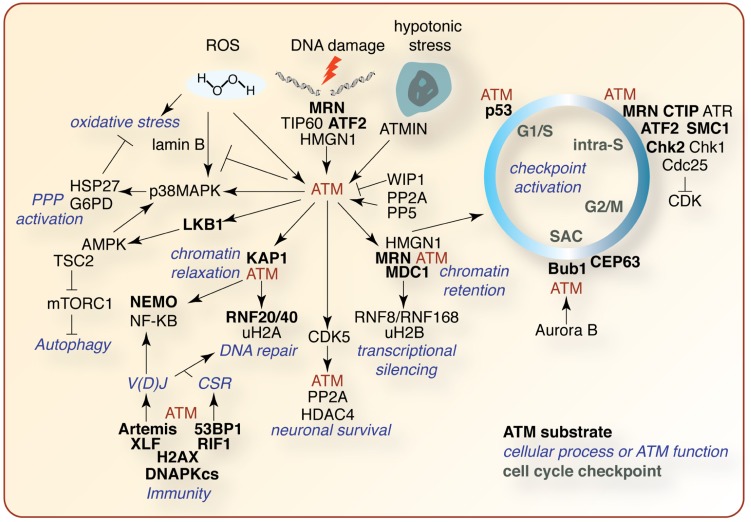
**Selected summary of the ATM signaling network**. A non-comprehensive schematic of ATM signaling pathways described in the text. ATM substrates are indicated in bold black, other pathway effectors in black, affected cellular processes in blue, and cell cycle checkpoints in green. See text for details regarding the signaling pathways depicted.

Our knowledge of ATM signaling in development and disease is ever expanding due to the creative work of dozens of labs using diverse experimental systems (**Figure 4**). The identification of specific ATM substrates and their functions will be instrumental in elucidating the mechanisms by which ATM can control so many critical cellular processes. This will require innovation at the level of sensitive high-throughput analysis of phosphorylation, and other PTMs, as well detailed single gene studies in available model systems. It is clear that the generation of animal models has been, and continues to be, invaluable to our understanding of ATM in tissue-specific processes such as apoptosis, CNS development, immunity, and angiogenesis, but they also do not faithfully recapitulate many crucial aspects of the human disease. Gene editing technologies, such as zinc-finger nucleases, should allow the manipulation of ATM and associated genes in higher organisms that may more faithfully recapitulate the human condition, particularly CNS pathology. Coupled with high-resolution sequencing and mass spectrometry-based technologies, including metabolic profiling, the pieces of the ATM puzzle will continue to fall into place and new strategies to exploit this knowledge can be used to benefit patients.

## Conflict of Interest Statement

The authors declare that the research was conducted in the absence of any commercial or financial relationships that could be construed as a potential conflict of interest.
